# Mouse myofibers lacking the SMYD1 methyltransferase are susceptible to atrophy, internalization of nuclei and myofibrillar disarray

**DOI:** 10.1242/dmm.022491

**Published:** 2016-03-01

**Authors:** M. David Stewart, Suhujey Lopez, Harika Nagandla, Benjamin Soibam, Ashley Benham, Jasmine Nguyen, Nicolas Valenzuela, Harry J. Wu, Alan R. Burns, Tara L. Rasmussen, Haley O. Tucker, Robert J. Schwartz

**Affiliations:** 1 Department of Biology and Biochemistry, University of Houston, Houston, TX 77204, USA; 2 Department of Computer Science and Engineering Technology, University of Houston-Downtown, Houston, TX 77002, USA; 3 Stem Cell Engineering Department, Texas Heart Institute at St Luke's Episcopal Hospital, Houston, TX 77030, USA; 4 College of Optometry, University of Houston, Houston, TX 77204, USA; 5 Department of Molecular Physiology, Baylor College of Medicine, Houston, TX 77030, USA; 6 Department of Molecular Biosciences, Institute for Cellular Molecular Biology, The University of Texas at Austin, Austin, TX 78712, USA

**Keywords:** Genetics, Development, Muscle, Myocyte, SMYD1, Methylation, Myopathy

## Abstract

The *Smyd1* gene encodes a lysine methyltransferase specifically expressed in striated muscle. Because *Smyd1-*null mouse embryos die from heart malformation prior to formation of skeletal muscle, we developed a *Smyd1* conditional-knockout allele to determine the consequence of SMYD1 loss in mammalian skeletal muscle. Ablation of SMYD1 specifically in skeletal myocytes after myofiber differentiation using *Myf6^cre^* produced a non-degenerative myopathy. Mutant mice exhibited weakness, myofiber hypotrophy, prevalence of oxidative myofibers, reduction in triad numbers, regional myofibrillar disorganization/breakdown and a high percentage of myofibers with centralized nuclei. Notably, we found broad upregulation of muscle development genes in the absence of regenerating or degenerating myofibers. These data suggest that the afflicted fibers are in a continual state of repair in an attempt to restore damaged myofibrils. Disease severity was greater for males than females. Despite equivalent expression in all fiber types, loss of SMYD1 primarily affected fast-twitch muscle, illustrating fiber-type-specific functions for SMYD1. This work illustrates a crucial role for SMYD1 in skeletal muscle physiology and myofibril integrity.

## Abstract (precis)

**Summary:** Elimination of the lysine methyltransferase SMYD1 from mouse skeletal muscle caused myopathy with excessive internal nuclei, atrophy, myofibrillar disorganization and broad upregulation of muscle gene expression.

## INTRODUCTION

The *SMYD1* gene encodes an evolutionarily conserved histone methyltransferase containing a catalytic SET domain split by a zinc-finger-rich MYND domain. The SET domain is required for trimethylation of histone H3 lysine 4 (H3K4me3) (Sirinupong et al., 2010; Tan et al., 2006), a chromatin modification associated with active transcription (Strahl et al., 1999). The MYND domain is a protein-protein interaction motif best known for its association with histone deacetylase (HDAC)-containing co-repressor complexes (Liu et al., 2007; Lutterbach et al., 1998; Masselink and Bernards, 2000). Thus, SMYD1 possesses both transcriptional activator and repressor properties. SMYD1 also localizes to the sarcoplasm, where it directly interacts with myosin through its C-terminal region (Just et al., 2011).

Expression of *SMYD1* is highly restricted to the striated muscles of human, fish, frog, chicken and mouse (Gottlieb et al., 2002; Kawamura et al., 2008; Li et al., 2009; Tan et al., 2006). *Smyd1* mRNA was detected in the earliest cardiac progenitor cells within the precardiac mesoderm. Later in embryonic development, its expression becomes restricted to the myocardium (Gottlieb et al., 2002; Rasmussen et al., 2015). In the skeletal muscle lineage, *Smyd1* mRNA is detectable within the somites as early as embryonic day 9.5 (E9.5); protein is first detectable within differentiating myoblasts (Nagandla et al., 2016). Expression persists in both cardiac and skeletal myocytes throughout development and into adulthood.

SMYD1 plays an essential role in heart and skeletal muscle development. *Smyd1*-null mice die *in utero* prior to skeletal muscle development, owing to heart defects (Gottlieb et al., 2002). Because of this early lethality, information regarding the *in vivo* skeletal muscle function(s) of SMYD1 has been limited to studies in zebrafish. Zebrafish have two highly similar genes, s*myd1a* and s*myd1b*. Knockdown of *smyd1b* results in disruption of myofibril formation in both skeletal and cardiac muscles (Li et al., 2013; Tan et al., 2006). Likewise, the zebrafish mutant *flatline* (*fla*), carrying a null mutation within *smyd1b*, fails to form sarcomeres (Just et al., 2011). Individual morpholino knockdowns of the two *smyd1* genes showed *smyd1b* to be more crucial of the two for myofibrillogenesis; however, ectopic expression of *smyd1a* can substitute for loss of *smyd1b* (Gao et al., 2014). The mammalian and zebrafish *Smyd1* genes encode two mRNA transcripts via inclusion or exclusion of a small exon encoding 13 amino acids (Hwang and Gottlieb, 1997). Although differential functions for these isoforms have not been addressed in mouse or human, the zebrafish protein encoded by the inclusion of the small exon localizes to the M-line, whereas the shorter isoform exhibits weak sarcomere association (Li et al., 2011). The precise function of SMYD1 in myofibrillogenesis has remained unclear regardless of species. SMYD1 associates with nascent myosin during sarcomerogenesis and then localizes to the M-line of mature sarcomeres (Just et al., 2011). Thick filament chaperones are upregulated in the absence of *smyd1b*, supporting the idea that SMYD1 stabilizes myosin (Li et al., 2013). Collectively, the published data concur that SMYD1 is a striated-muscle-specific methyltransferase essential for proper development of cardiac and skeletal muscle, and which might stabilize assembly of the contractile apparatus. Thus, further investigation of the molecular biology of SMYD1 is essential to our understanding of the molecular genetics of muscle physiology.

To circumvent embryonic lethality in mice, we generated a *Smyd1* conditional-knockout (CKO) allele (Rasmussen et al., 2015) to test the hypothesis that SMYD1 is required for proper skeletal muscle development in mammals. Specific inactivation of *Smyd1* in skeletal myocytes resulted in a myopathy with excessive internal nuclei, atrophy and myofibril disarray. The pathology was primarily manifest in fast-twitch muscles, with males being more severely affected than females. These phenotypic findings along with gene-expression profiling suggest a mechanism by which myofibers fail to fully mature. That SMYD1 also regulates cardiac development and function opens the possibility that *SMYD1* is a genetic link between skeletal and cardiac myopathies.

## RESULTS

To determine the function of SMYD1 in skeletal muscle, we conditionally inactivated the *Smyd1* gene using *Myf6^cre^*. In this strain, an internal ribosome entry site (*IRES*)*-Cre* cassette is located within the 3′ untranslated region (UTR) of the *Myf6* gene, allowing bicistronic expression of *Myf6* and *Cre* (Keller et al., 2004). Our genetic approach included the *Rosa26^YFP^* allele, which allowed us to monitor cells derived from *Myf6^cre^*-expressing cells. By monitoring yellow fluorescent protein (YFP) fluorescence, we found the onset of *Myf6^cre^*-induced recombination to occur at E17.5, being just barely detectable in skeletal muscles at this point. Strong YFP fluorescence was not observed until the day of birth (data not shown). *Smyd1* is first expressed in the somitic myoblasts as early as E9.5 and expression persists throughout myogenesis and in mature myofibers (Gottlieb et al., 2002; Nagandla et al., 2016). Thus, SMYD1 might have roles in both myogenesis and myofiber homeostasis. Using *Myf6^cre^*, we specifically studied the role of SMYD1 in myofibers post-myogenesis. Using 6-week-old mice, we confirmed that *Myf6^cre^* induces recombination of *loxP* sites in all muscles examined (Fig. S1). All experiments were performed and tissues collected at 6 weeks of age unless otherwise noted.

### *Smyd1* CKO mice are smaller with reduced strength

**Fig. 1.**
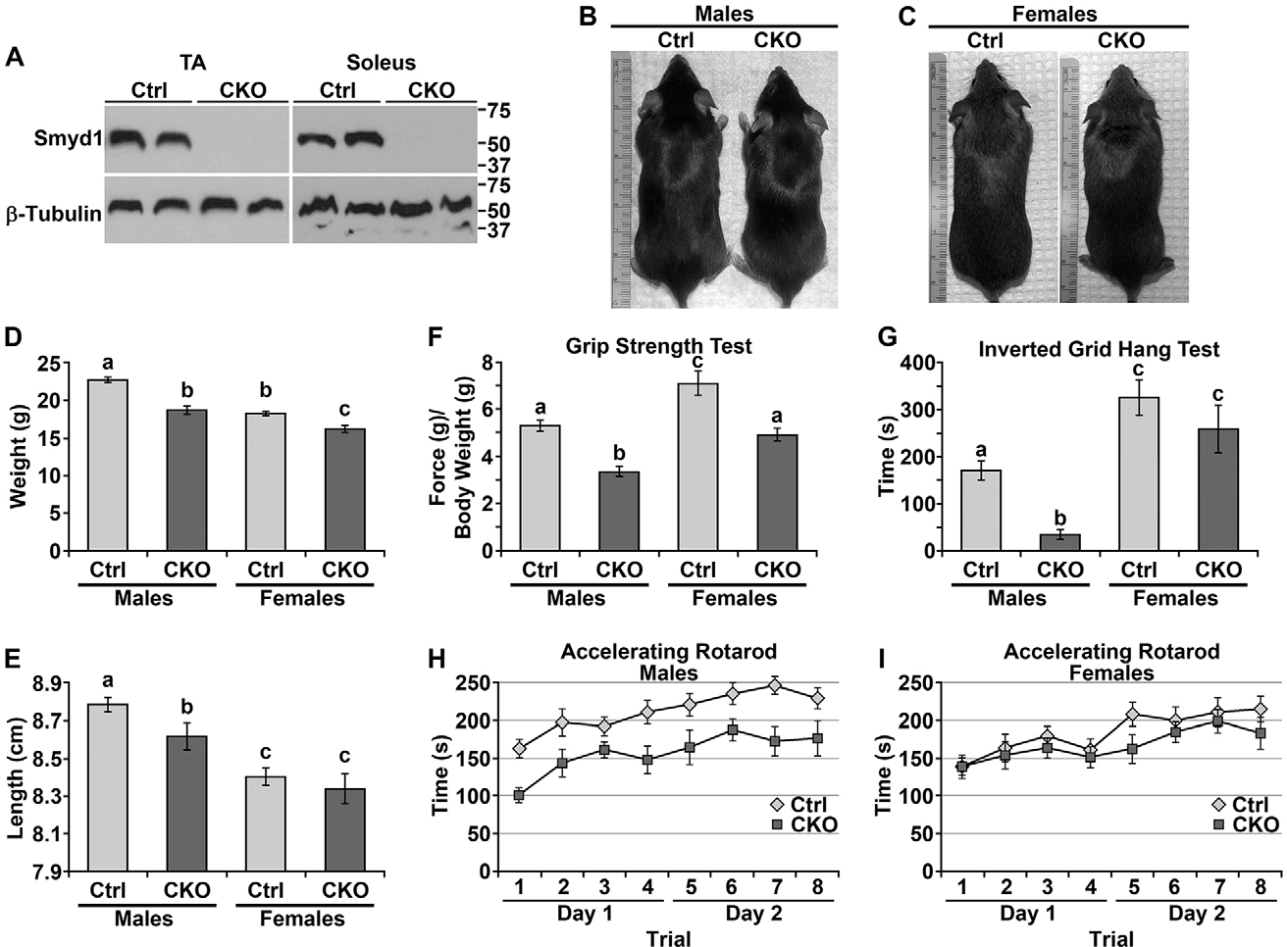
***Smyd1* CKO mice are smaller with reduced strength.** (A) Western blots showing the absence of SMYD1 protein in CKO muscles. (B,C) *Smyd1* CKO males appeared slightly smaller than controls. Female CKOs appeared similar to controls. (D) *Smyd1* CKO mice weighed less than controls. *n*≥17 animals/group. (E) Body length (naso-anal length) was reduced for *Smyd1* CKO males, but not females. *n*≥17 animals/group. (F,G) *Smyd1* CKO mice were weaker than controls. (F) Four-paw grip-strength tests. Data are maximum force of three pulls per animal normalized to body weight. *n*≥8 animals/group. (G) Inverted grid hang tests. Data are the length of time the mouse is able to hang from the grid before falling. *n*≥13 animals/group. (D-G) Statistics were ANOVA+post hoc tests. Significant differences (*P*<0.05) are denoted by the lowercase letters above each bar. (H,I) Accelerating rotarod tests for males (H) and females (I). Mice of all groups progressively increased the length of time they could run on the rotarod (two-way ANOVA, *P*<0.001 for both males and females). Male and female CKOs underperformed their control counterparts (two-way ANOVA; males: *P*<0.0001, *n*≥12 animals/group; females: *P*=0.025, *n*≥13 animals/group); however, the mean difference in elapsed time for male controls vs male CKOs (55.1±7.7 s) was much greater than that for female controls vs female CKOs (17.8±7.9 s). All data was obtained at 6 weeks of age. Error bars show s.e.m. Ctrl, control; CKO, conditional knockout; TA, tibialis anterior.

Western blots were performed to confirm loss of SMYD1 protein in CKO muscles. SMYD1 protein levels were undetectable in both the tibialis anterior and soleus of CKO mice (Fig. 1A). Visually, male CKOs appeared smaller than control littermates. Female CKOs appeared only marginally smaller than control littermates (Fig. 1B,C). Body weight was reduced for both male and female CKO mice compared with controls (Fig. 1D). Males, but not females, exhibited reduced body length (naso-anal length) (Fig. 1E).

Suspecting some form of myopathy in *Smyd1* CKO mice, we performed several experiments to assay strength. A four-paw grip strength test revealed weakness for both CKO males and females (Fig. 1F). We assayed relative strength/endurance with an inverted grid hanging test, which measures the length of time that the mouse can cling upside down before falling. Male CKOs underperformed controls in this test. In contrast, female CKOs performed equal to controls (Fig. 1G). Last, we performed accelerating rotarod tests, which assay strength, motor coordination and learning (males, Fig. 1H; females, Fig. 1I). With successive trials, mice of all groups progressively increased the length of time they could run on the rotarod. Male and female CKOs underperformed their control counterparts; however, the mean difference in elapsed time for male controls vs male CKOs (55.1±7.7 s) was much greater than that for female controls vs female CKOs (17.8±7.9 s). Collectively, these results revealed loss of strength and reduced endurance in the absence of SMYD1, which more severely affected male mice.

### *Smyd1* CKO mice exhibited reduced muscle mass

**Fig. 2.**
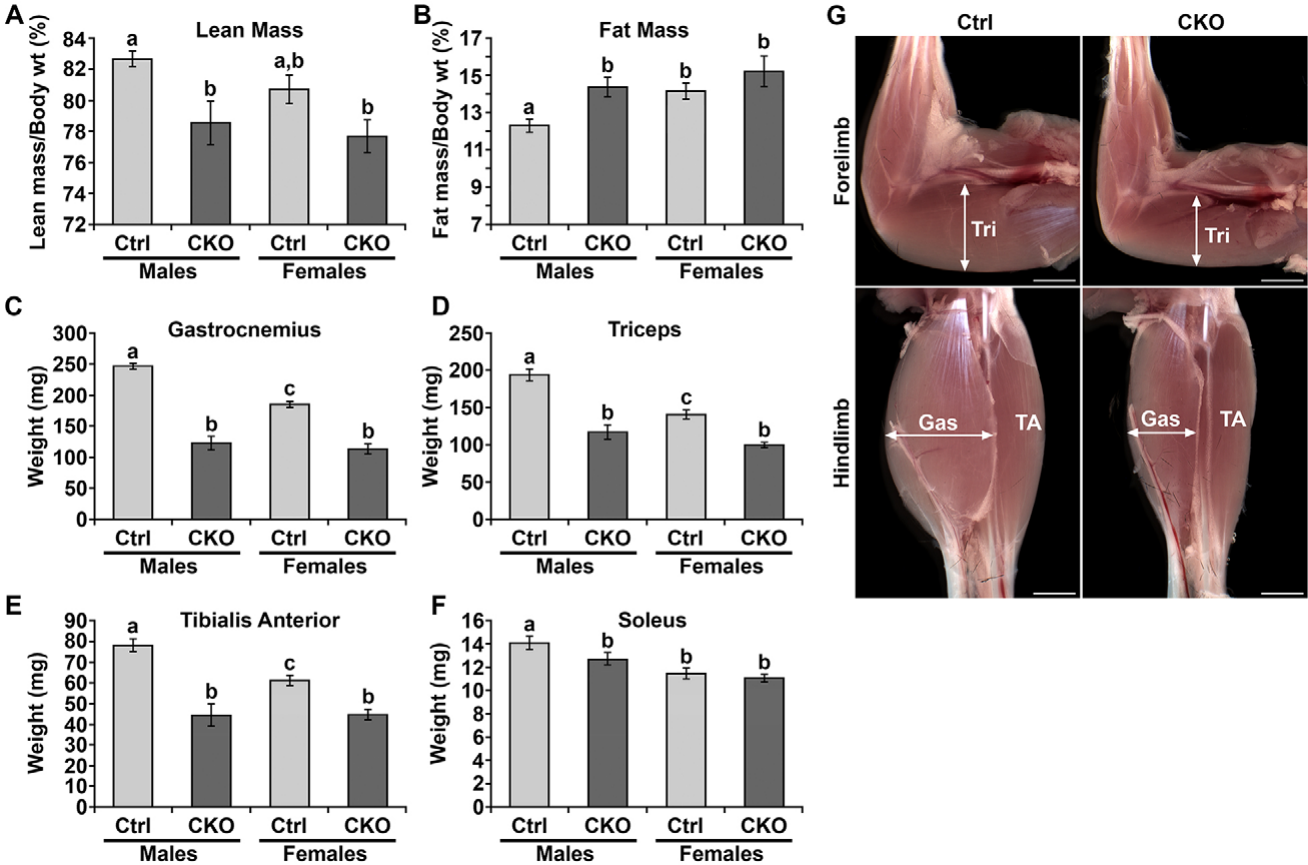
***Smyd1* CKO mice exhibited reduced muscle mass.** (A,B) Body composition analysis by MRI (*n*≥12 animals/group). (A) Percent lean mass was reduced in *Smyd1* CKO males and females. (B) Percent fat mass was increased in *Smyd1* CKO males and females. (C-F) Wet weight of individual muscles (weighed in pairs). For males, muscle mass was reduced for all muscles examined. For females, muscle mass was reduced for all except the soleus (*n*≥10 animals/group for gastrocnemius, soleus and triceps; *n*≥6 animals/group for tibialis anterior). (G) Stereoimages of the muscles of the forelimbs and hindlimbs (males only). Scale bars: 2 mm. All data was obtained at 6 weeks of age. Statistics were ANOVA+post hoc tests. Significant differences (*P*<0.05) are denoted by the lowercase letters above each bar. Error bars show s.e.m. Ctrl, control; CKO, conditional knockout; Gas, gastrocnemius; TA, tibialis anterior; Tri, triceps.

Body composition was assayed by magnetic resonance imaging (MRI) and showed reduced lean mass and increased fat mass for both CKO males and females compared with sex-matched controls (Fig. 2A,B). Individual muscles, comprising a range of fiber-type compositions (tibialis anterior, fast twitch; soleus, slow twitch; gastrocnemius and triceps, mixed), were dissected and weighed. In males, we observed reduced mass for all muscles studied. For females, a significant difference in mass was observed for gastrocnemius, triceps and tibialis anterior, but not soleus (Fig. 2C-F). At the gross morphological level, *Smyd1* CKO muscles appeared smaller than those of control mice (males, Fig. 2G; females, Fig. S2).

### High percentage of fibers with central nuclei in fast-twitch muscle

**Fig. 3.**
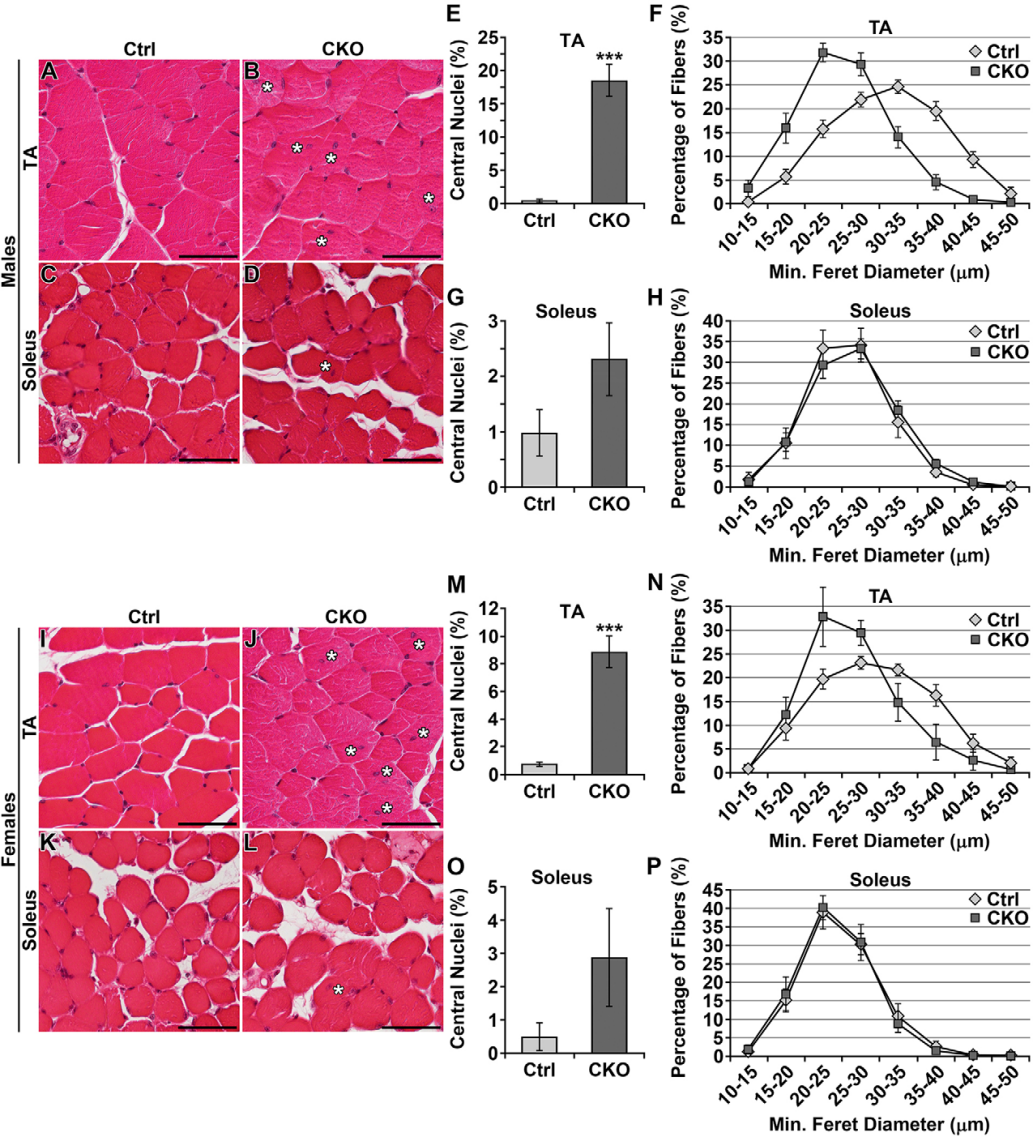
**High percentage of fibers with central nuclei in fast-twitch muscle.** (A-H) 6-week-old males. (A-D) Central nuclei were prevalent in the *Smyd1* CKO tibialis anterior. White asterisks (*) indicate central nuclei. (E) Percentage of tibialis anterior myofibers with central nuclei (*n*≥6 animals/group). (F) Distribution of myofiber minimum Feret diameter in the tibialis anterior. *Smyd1* CKO tibialis anterior had increased numbers of hypotrophic fibers (ANOVA, *P*<0.05; *n*≥6 animals/group). (G) Percentage of myofibers with central nuclei in the soleus. The difference was not significant (*n*≥7 animals/group). (H) Distribution of myofiber minimum Feret diameter in the soleus. No difference was observed (*n*≥7 animals/group). (I-P) 6-week-old females. (I-L) Central nuclei were prevalent in the CKO tibialis anterior, but to a lesser extent than observed for CKO males. (M) Percentage of tibialis anterior myofibers with central nuclei (*n*=6 animals/group). (N) Distribution of myofiber minimum Feret diameter in the tibialis anterior. *Smyd1* CKO tibialis anterior had increased numbers of hypotrophic fibers (ANOVA, *P*<0.05; *n*=6 animals/group). (O) Percentage of myofibers in the soleus with central nuclei. The difference was not significant (*n*≥10 animals/group). (P) Distribution of myofiber minimum Feret diameter in the soleus. No difference was observed (*n*≥10 animals/group). ***P<0.001, Student's *t*-test. Error bars show s.e.m. Scale bars: 50 μm. Ctrl, control; CKO, conditional knockout; TA, tibialis anterior.

We next examined the consequence of *Smyd1* deficiency on the histology of a number of muscles/muscle groups (triceps, quadriceps, gastrocnemius, tibialis anterior and soleus) in hematoxylin and eosin (H&E)-stained sections. Representative images from male and female tibialis anterior and soleus are shown in Fig. 3A-D,I-L and for triceps, quadriceps and gastrocnemius in Fig. S3. Although no necrotic or degenerating fibers were apparent, we observed a large number of fibers with central/internal nuclei and hypotrophic fibers. Quantification of the percentage of fibers with central nuclei in the tibialis anterior (fast twitch) and soleus (slow twitch) at 6 weeks of age revealed that, in male mice, 19% of fibers in the *Smyd1* CKO tibialis anterior exhibited central nuclei as compared to 0.8% of fibers in controls (Fig. 3E). Compared to controls, female *Smyd1* CKOs also exhibited increased numbers of fibers with central nuclei in the tibialis anterior (9% in CKOs vs 0.8% in controls) (Fig. 3M). Note that the percentage of fibers with central nuclei in female CKOs was less than that for male CKOs (males, 19%; females, 9%).

Myofiber size was quantified by measuring minimum Feret diameter. The tibialis anterior of *Smyd1* CKO male mice exhibited increased numbers of hypotrophic fibers as compared to control littermates (mean min. Feret diameter: Ctrl, 31.18±0.80 μm; CKO, 25.15±0.83 μm; ANOVA, *P*<0.05) (Fig. 3F). Female *Smyd1* CKO mice also exhibited myofiber hypotrophy in the tibialis anterior (mean min. Feret diameter: Ctrl, 29.70±1.34 μm; CKO, 26.44±1.71 μm; ANOVA, *P*<0.05) (Fig. 3N). Consistent with our strength, muscle mass and central nuclei data, the percent reduction in fiber size was less for *Smyd1* CKO females than for males (19% for males vs 11% for females). In neither males nor females was the soleus significantly affected by this myopathy as judged by % central nuclei (Fig. 3G,O) or minimum Feret diameter (mean min. Feret diameter: Ctrl males, 25.72±1.05 μm; CKO males, 26.45±0.65 μm; Ctrl females, 29.70±1.34 μm; CKO females, 26.44±1.71 μm; ANOVA, *P*>0.05) (Fig. 3H,P). Thus, the major pathological features primarily affect fast-twitch muscle. Furthermore, these data indicate a gender-specific effect in which males are more severely affected than females. Because males displayed a consistent strong phenotype, the remainder of the experiments were conducted using only male tissues.

To determine whether disease worsened with age, we allowed a group of male mice to age to 6 months. Disease progression was little changed for the tibialis anterior but increased in the soleus. We observed high numbers of hypotrophic fibers and fibers with central nuclei in the tibialis anterior (Fig. S4A,B). In contrast to younger (6-week-old) CKOs, the soleus now showed increased numbers of fibers with central nuclei (Fig. S4C,D). For the tibialis anterior, the percentage of fibers with centralized nuclei was 21% (Fig. S4E), similar to that observed at 6 weeks. Unlike 6-week-old mice, *Smyd1* CKO soleus exhibited significantly more fibers with central nuclei than controls, albeit a modest increase. *Smyd1* CKO soleus exhibited 4% of fibers with central nuclei, whereas controls exhibited less than 1% (Fig. S4G). For tibialis anterior, average fiber minimum Feret diameter for control mice increased with age to 41.32±1.64 μm, as compared to 30.96±0.54 μm for CKO mice – a value similar to that observed for CKOs at 6 weeks (ANOVA, *P*<0.05) (Fig. S4F). For the soleus we observed no significant difference in fiber size distribution (mean min. Feret diameter: Ctrl, 32.55±0.79 μm; CKO 31.04±0.72 μm; ANOVA, *P*>0.05) (Fig. S4H).

### Absence of regenerating myofibers and normal sarcolemma in *Smyd1* CKO muscle

**Fig. 4.**
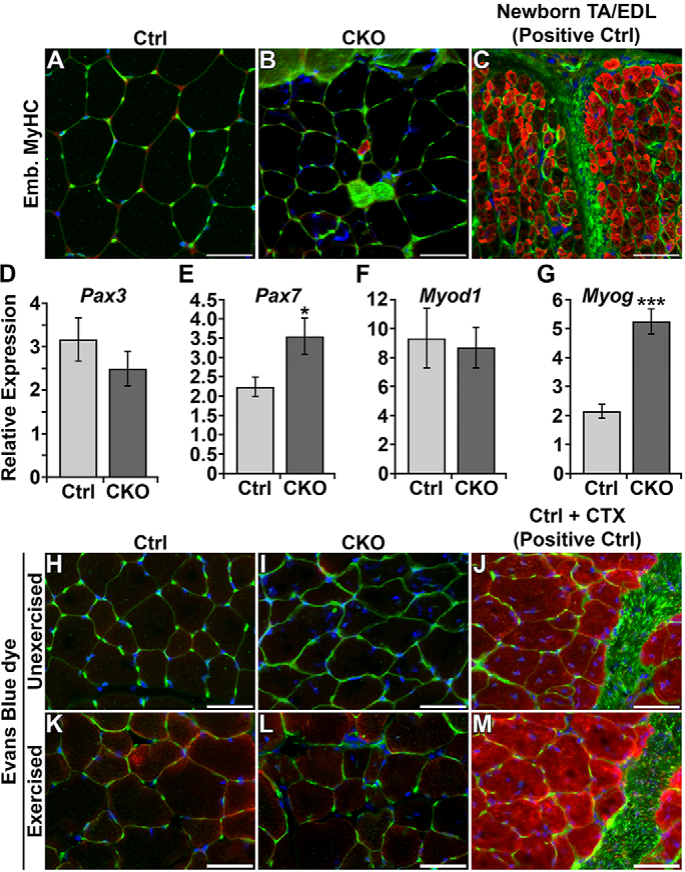
**Absence of regenerating myofibers and normal sarcolemma in *Smyd1* CKO muscle.** (A-C) Near complete lack of embryonic myosin heavy chain (eMyHC)-positive myofibers in the *Smyd1* CKO. Red, eMyHC; green, sarcolemma (WGA-488); blue, nuclei (DAPI). No eMyHC-positive myofibers were found in control animals (*n*=3) (A). Only a single eMyHC-positive myofiber was found in *Smyd1* CKO tibialis anterior (*n*=3) (B). Newborn (postnatal day 1) hindlimb muscles were used as a positive control for eMyHC (C). (D-G) Results of real-time PCR assays for regeneration-associated genes (*n*=6 animals/group). Expression of *Pax3* (D) and *Myod1* (F) were unchanged. Expression of *Pax7* (E) and *Myog* (G) were increased in the *Smyd1* CKO. (H-M) No evidence for impaired sarcolemmal integrity in the *Smyd1* CKO. Red, Evans blue dye; green, sarcolemma (WGA-488); blue, nuclei (DAPI). Uptake of Evans blue dye in unexercised (H,I) or exercised (K,L) mice (*n*=3 animals/group). Cardiotoxin-treated muscle was used as a positive control (J,M). All assays were performed using tibialis anterior muscle from 6-week-old male mice. *P<0.05, ***P<0.001, Student's *t*-test. Error bars show s.e.m. Scale bars: 50 μm. Ctrl, control; CKO, conditional knockout; CTX, cardiotoxin; EDL, extensor digitorum longus; TA, tibialis anterior.

To determine whether the myofibers with central nuclei were the result of regeneration, we assayed the number of regenerating fibers by immunolocalization of embryonic myosin heavy chain (eMyHC). The tibialis anterior of control mice exhibited no eMyHC-positive fibers (Fig. 4A). Only a single small eMyHC positive fiber was found in the three CKOs assayed (Fig. 4B). Thus, eMyHC-marked regenerating fibers do not correlate with the presence of central nuclei. Furthermore, the extremely low numbers of regenerating fibers cannot account for the 20% of fibers with central nuclei. Newborn (postnatal day 0) hindlimb, which is rich in eMyHC-positive fibers, was used as a positive control (Fig. 4C). We next assayed a representative panel of regeneration genes for alterations in transcript expression. Expression of *Pax3* and *Myod1* was unaffected (Fig. 4D,F), whereas expression of *Pax7* and *Myog* was modestly increased (Fig. 4E,G).

Sarcolemmal integrity in the tibialis anterior was assayed by Evans blue dye uptake. No Evans-blue-dye-positive fibers were identifiable in control or *Smyd1* CKO mice that were unexercised (Fig. 4H,I) or subjected to a single period of treadmill exercise (30 min at 12 m/min, uphill 5°) (Fig. 4K,L). As a positive control, we observed widespread Evans-blue-dye-positive fibers in control mice that received a single intramuscular injection of cardiotoxin 13 days earlier (Fig. 4J,M). Thus, sarcolemmal integrity is intact in *Smyd1* CKO myofibers, which is indicative of myopathy rather than muscular dystrophy.

Collectively, these data indicate very low levels of regeneration, and normal sarcolemmal integrity, in the absence of SMYD1. These minor increases in regeneration markers cannot account for the large numbers of fibers with central nuclei. These data coupled with the absence of necrotic or degenerating fibers suggest that the myofibers with internal nuclei have failed to fully mature rather than being the result of *de novo* regeneration.

### Increased numbers of oxidative myofibers

**Fig. 5.**
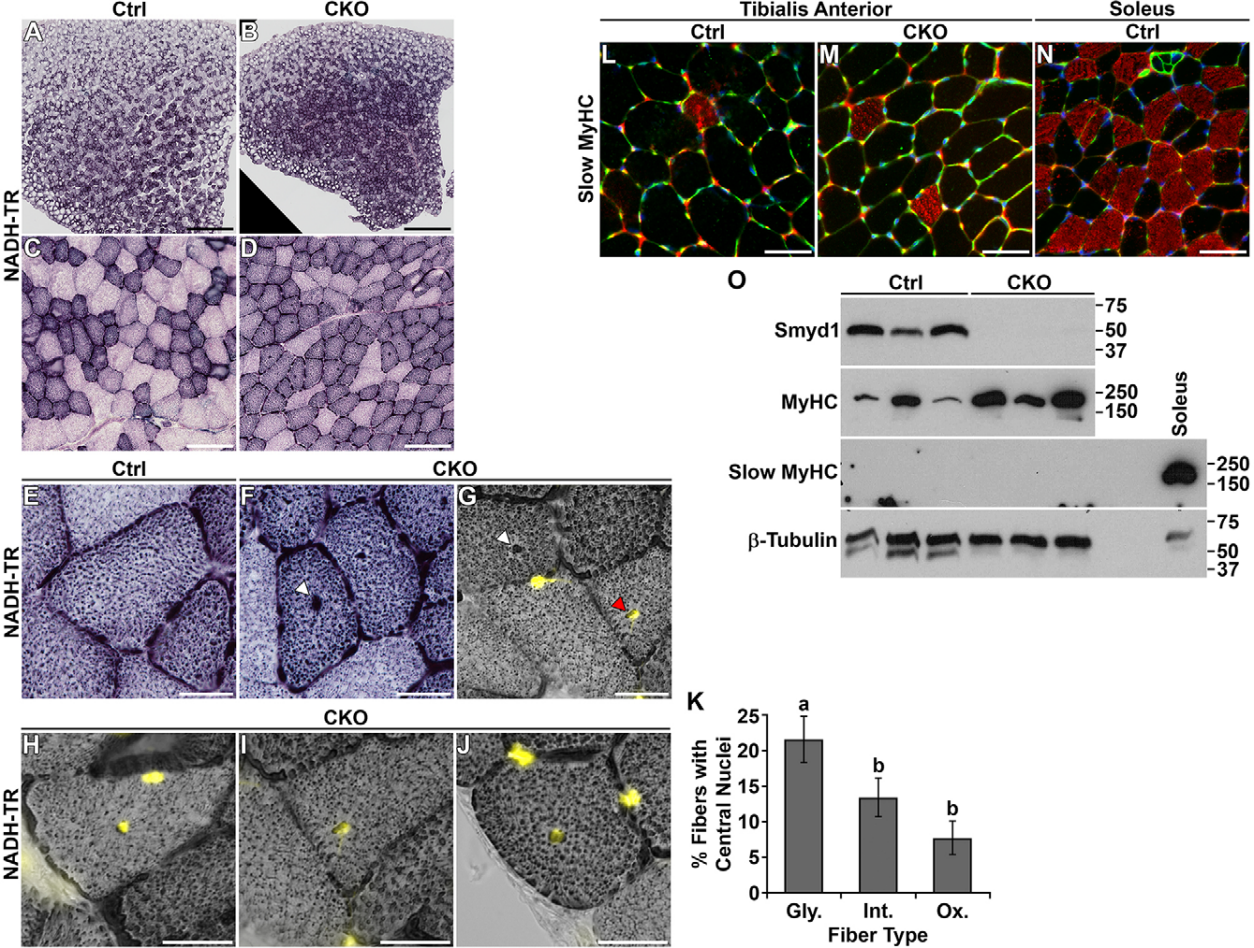
***Smyd1* CKO mice exhibited increased numbers of oxidative myofibers.** (A-J) NADH-TR staining of the tibialis anterior. *Smyd1* CKOs had more fibers with intense oxidative stain (A-D). The only intermyofibrillar network irregularity observed was a central dense focus of oxidative stain (E-G). The dense foci frequently correlated with centralized nuclei [yellow, nuclei (DAPI); white arrowhead, foci of oxidative stain; red arrowhead, foci of oxidative stain that colocalizes with a central nucleus] (G). Centralized nuclei were observed in glycolytic (H), intermediate (I) and oxidative (J) fibers. (K) Centralized nuclei were most abundant in glycolytic fibers (CKOs only, *n*=5). Fibers were classified as glycolytic (Gly.), intermediate (Int.) or oxidative (Ox.) based on staining intensity. (L-N) There was no difference in the percentage of type I fibers in the tibialis anterior of control (L) and CKO (M) mice. Soleus was used as a positive control (N). Red, slow MyHC; green, sarcolemma (WGA-488); blue, nuclei (DAPI). (O) Western blots using extracts of tibialis anterior (one animal/lane). SMYD1 protein was undetectable in CKO muscle. Total MyHC increased in the CKO. Slow MyHC was undetectable in both control and CKO tibialis anterior; soleus was included as a positive control. Scale bars: 500 μm (A,B), 100 μm (C,D), 25 μm (E-J) and 50 μm (L-N). All data were obtained from 6-week-old males. Statistics were ANOVA+post-hoc tests. Significant differences (*P*<0.05) are denoted by the lowercase letters above each bar. Error bars show s.e.m. Ctrl, control; CKO, conditional knockout; MyHC, myosin heavy chain.

The prevalence of internal myonuclei in the absence of regenerating fibers is reminiscent of human centronuclear myopathy (CNM). CNM is typically associated with type I fiber predominance and, in the case of *DNM2-* and *BIN1-*related CNM, a radial intermyofibrillar network (Böhm et al., 2014; Chen et al., 2015; Cowling et al., 2014; Romero, 2010; Romero and Bitoun, 2011). To study these characteristics, we performed NADH-tetrazolium reductase (NADH-TR) staining on frozen sections of tibialis anterior. We observed increased prevalence of oxidative fibers in the *Smyd1* CKO (Fig. 5A-D). At high magnification, we found no evidence for radial orientation of the intermyofibrillar network. The only irregularity observed was a central dense focus of oxidative stain (Fig. 5E,F). These dense foci generally, but not always, correlated with centralized nuclei (Fig. 5G). Centralized nuclei were observed in glycolytic, intermediate and oxidative fibers (Fig. 5H-J); however, they were most abundant in glycolytic fibers (Fig. 5K).

Abundance of type I fibers was assayed by immunofluorescence. We found no significant difference in the percentage of type I fibers in the tibialis anterior for controls and *Smyd1* CKOs (Ctrl, 0.51±0.89%; CKO, 3.83±5.18%; *n*=7/group) (Fig. 5L,M). Soleus served as a positive control (Fig. 5N). These results were confirmed by the virtually undetectable levels of slow MyHC in tibialis anterior western blots (Fig. 5O). Interestingly, total MyHC levels were increased in *Smyd1* CKOs. Collectively, these data suggest increased fast-oxidative fiber abundance in the absence of SMYD1, but very little fast-to-slow fiber-type switching.

### Abnormal myofibrillar architecture in the absence of SMYD1

**Fig. 6.**
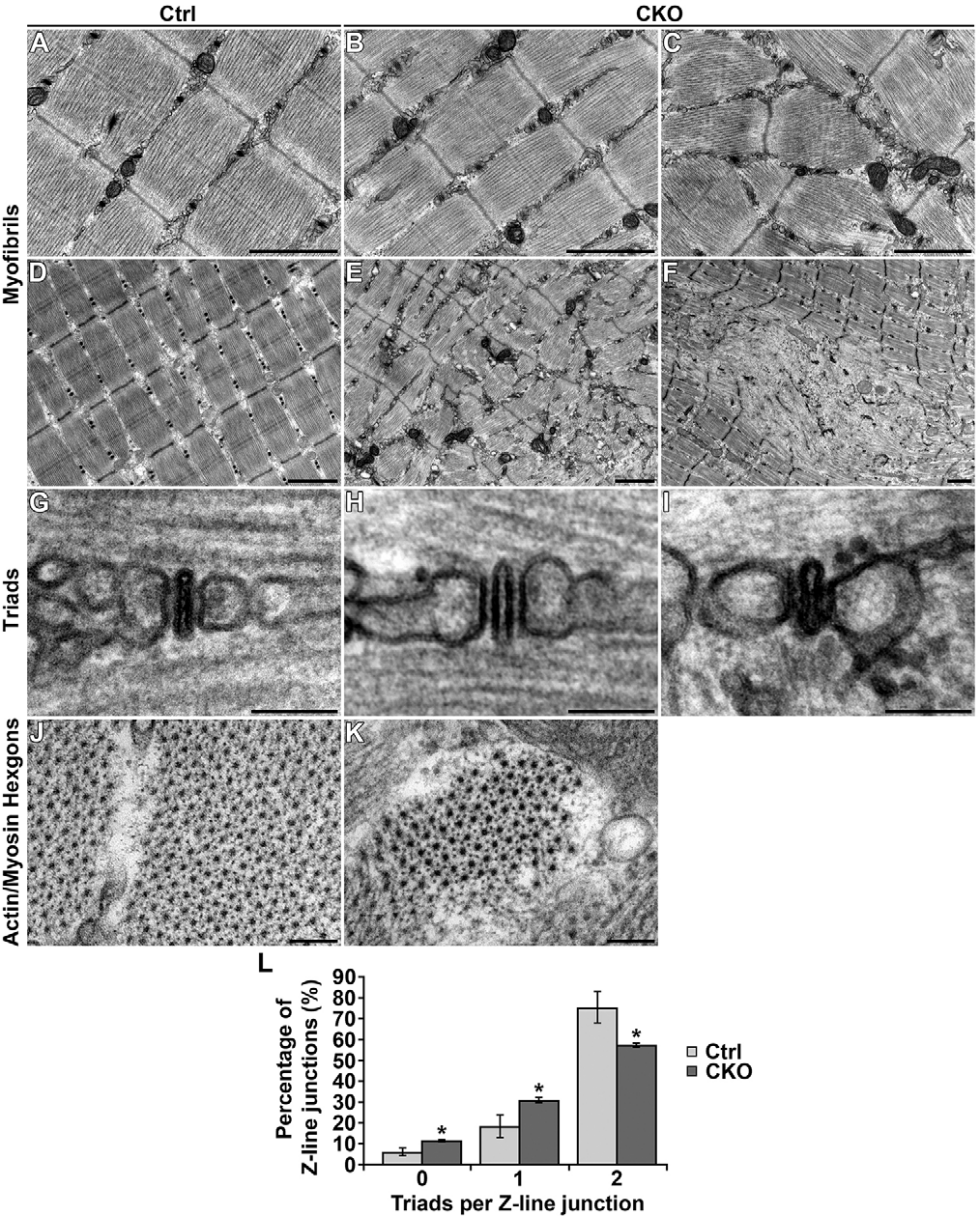
**Abnormal myofibrillar architecture in the absence of SMYD1.** (A-K) Ultrastructure images of the tibialis anterior. Control mice exhibited normal myofibrillar architecture (A,D). *Smyd1* CKO muscles showed regions of normal ultrastructure (B), areas of myofibrillar disorganization (C,E) and foci of sarcomere disassembly (F). (G-I) Triads appeared normal regardless of whether they were located in normal regions (H) or regions of disorganization (I). (J,K) *Smyd1* CKO muscles exhibited normal actin/myosin hexagons in transverse sections. (L) *Smyd1* CKO muscle had reduced numbers of triads. Data are the number of Z-lines flanked by zero, one or two triads. *Smyd1* CKO muscles had increased numbers of Z-lines surrounded by zero or one triad and decreased numbers of Z-lines bordered by two triads (*n*=3 animals/group; *P<0.05, ANOVA+post-hoc tests). Error bars show s.e.m. Data were obtained from 6-week-old male mice. Scale bars: 1 μm (A-F), 50 nm (G-I) and 100 nm (J,K). Ctrl, control; CKO, conditional knockout.

The most notable ultrastructure abnormality was regional disorganization of myofibrils. Ultrastructure was assayed by transmission electron microscopy (TEM) using sections from the tibialis anterior. Control mice exhibited uniform evenly spaced parallel sarcomeres (Fig. 6A,D). *Smyd1* CKO tibialis anterior exhibited many regions where myofibrillar architecture appeared normal (Fig. 6B); however, regions of antiparallel sarcomeres (Fig. 6C) or general disorganization (Fig. 6E) were not uncommon. Foci of complete sarcomere disassembly were also found (Fig. 6F). Because these foci are visible in Toluidine-blue-stained sections under the light microscope, we were able to measure their area and abundance. The foci ranged in size from 30.4 to 1062.7 μm^2^ and covered 2.4±1.1% of total longitudinal area. The average size of a single focus was 271.5±29.2 μm^2^. Data were derived from 96 measurements from three animals (>27 measurements/animal). Actin/myosin hexagons appeared normal in all transverse sections (Fig. 6J,K).

Human CNM is linked to impaired excitation-contraction coupling as a result of irregular triad structure or reduced numbers of triads (Jungbluth and Gautel, 2014; Romero and Bitoun, 2011). Control mice exhibited vast numbers of triads with correct appearance (Fig. 6G). In *Smyd1* CKO muscle, no obvious abnormalities were noted in triad structure regardless of whether the triad was located in a region with normal myofibrillar architecture (Fig. 6H) or a region of disorganized myofibrils (Fig. 6I). However, *Smyd1* CKO mice exhibited reduced numbers of triads as measured by counting the number of triads that flanked each Z-line. Normally, two triads flank each Z-line at the myofibril border. *Smyd1* CKO mice had fewer junctions with two triads and increased junctions with none or a single triad compared with controls (Fig. 6L). These data indicate intact triad structure but reduced complexity of the triad network in the absence of SMYD1.

### Gene expression alterations were specific to fast-twitch muscle

Looking for a connection between SMYD1 and CNM-associated pathways, we quantified transcript levels for the six major genes linked to CNM by real-time PCR (Table 1). In the tibialis anterior, we found upregulation of *Ryr1* and downregulation of *Ptpla*. *Bin1*, *Dnm2*, *Mtm1* and *Mtmr14* were unchanged. None of these genes showed altered expression in the soleus.

**Table 1.**
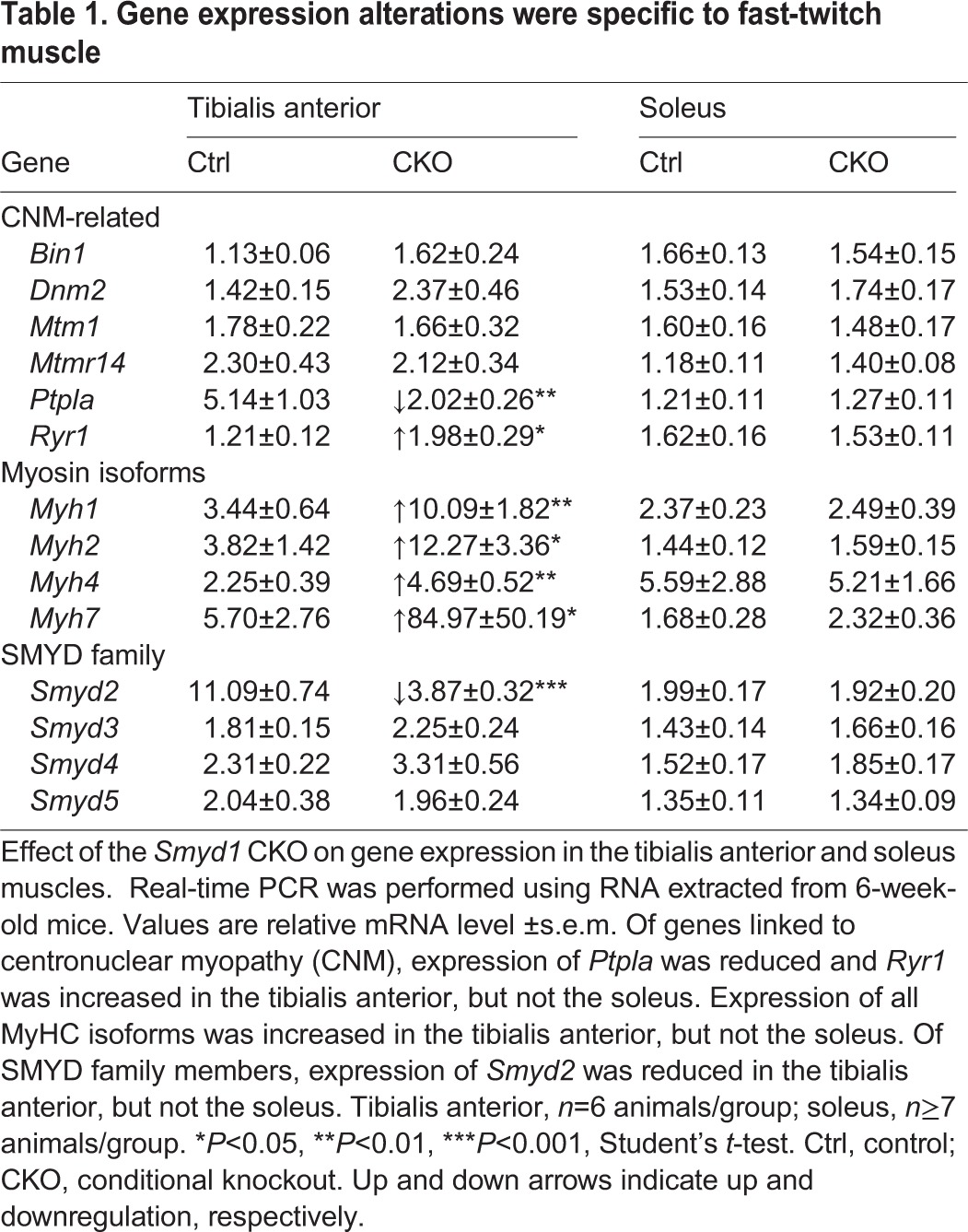
**Gene expression alterations were specific to fast-twitch muscle**

Because we found total MyHC protein levels to be elevated in the CKO (Fig. 5O), we assayed mRNA levels for the major MyHC isoforms (Table 1). Gene expression of all MyHC isoforms (*Myh1*, *2*, *4*, *7*) was increased in the tibialis anterior, but not in the soleus. These data further support the notion that the myopathy primarily affects fast-twitch muscle.

Unlike what we observed in the mouse, zebrafish morpholino or natural *smyd1a/b* mutants exhibit very severely impaired sarcomerogenesis (Just et al., 2011; Li et al., 2013; Tan et al., 2006). Thus, we reasoned that other SMYD family members might compensate for the loss of *Smyd1* in mice. To this end, we assayed expression of *Smyd2*, *3*, *4* and *5* (all known SMYD family members) (Table 1). We found *Smyd2* expression to be downregulated in the *Smyd1* CKO tibialis anterior. Expression of *Smyd3*, *4* and *5* was unaffected. No gene expression alterations were found in the soleus. Because no other SMYD family member was upregulated in response to the absence of SMYD1, it is unlikely that other SMYD family members compensate for the loss of SMYD1 in mice.

Our data consistently revealed a myopathy that primarily affected fast-twitch muscle. One explanation for this is that *Smyd1* might not be expressed in slow-twitch fibers, as was reported in zebrafish embryos (Just et al., 2011). Thus, we tested whether *Smyd1* is expressed in type I and II fibers by immunofluorescence using longitudinal sections of the extensor digitorum longus (EDL; which was used for ease of obtaining longitudinal sections) and soleus. Slow MyHC^+^ fibers were abundant in the soleus (Fig. S5A), but extremely rare in the EDL (Fig. S5B). SMYD1 showed sarcoplasmic localization in all fibers of both muscles (Fig. S5C,D). Next, we compared *Smyd1* mRNA and protein levels in the tibialis anterior and soleus by real-time PCR and western blot, respectively. The tibialis anterior and soleus showed comparable levels of *Smyd1* mRNA (Fig. S5G). SMYD1 protein levels were slightly higher in the tibialis anterior compared to the soleus (Fig. S5H). Thus, in mice, *Smyd1* is expressed in all fiber types. Furthermore, no other SMYD family members are upregulated to compensate for the loss of *Smyd1* in the soleus (Table 1). These data indicate that, despite SMYD1's presence in all fiber types, its absence produces defects that primarily manifest within type II fibers.

### Genes involved in muscle development are upregulated in *Smyd1* CKO muscle

To gain better insight into how loss of SMYD1 affected the myocyte transcriptome, we compared global gene expression in control versus *Smyd1* CKO tibialis anterior by RNA sequencing (RNA-seq). Expression of 603 genes was significantly upregulated (Fig. S6A) and expression of 630 genes was significantly downregulated (Fig. S6B) in the *Smyd1* CKO (cutoff was *P*<0.05). The top ten gene ontology terms associated with upregulated genes were primarily composed of muscle development pathways (Fig. S6C). Of the 42 genes upregulated in muscle gene ontology categories, 14 encode myofibrillar proteins and five encode regeneration-associated transcription factors. Also upregulated were ion channels, neuromuscular junction proteins, membrane structural components and signal transduction molecules. A heat map of upregulated genes associated with muscle gene ontology terms is presented in Fig. S7. The top ten gene ontology terms associated with downregulated genes mostly fell into the category of energy metabolism (Fig. S6D). Very few genes associated with fibrosis or inflammation exhibited altered expression in *Smyd1* CKO muscle, supporting the idea that this is not a degenerative muscle disease (Fig. S8). Because CNM is usually associated with impaired structure or function of the excitation-contraction coupling apparatus, we scrutinized the gene expression data for differentially expressed genes associated with T-tubules, membrane dynamics or calcium channels and found a large number of genes misregulated in these categories (Fig. S9). Very few of these genes encode actual triad components or are direct facilitators of calcium handling in myofibers; therefore, it is unclear whether this represents a meaningful issue with triad function. The results of the RNA-seq were consistent with that obtained by real-time PCR (Table S1). Collectively, our gene expression profiling supports the idea that *Smyd1* CKO myofibers fail to extinguish their developmental gene program or express a low level of regenerative gene activity in response to regional myofibrillar damage.

## DISCUSSION

Collectively, our data indicate that myofibers lacking SMYD1 are susceptible to atrophy, weakness, myofibrillar disarray and accumulation of internal nuclei. Broad upregulation of muscle gene expression and internal nuclei in the absence of myofiber regeneration suggests a non-degenerative repair process in response to myofibrillar damage. This phenotype has similarities to human CNM. CNM comprises a heterogeneous collection of similar diseases, all of which result in a high percentage of myofibers with central nuclei in the absence of degenerating or regenerating myofibers. Because *Smyd1* heterozygous mice are phenotypically normal, myopathies due to *Smyd1* mutations would likely be autosomal recessive. Autosomal recessive CNM has been linked to mutations in the genes encoding the membrane-deforming protein amphiphysin 2 (*BIN1*), the major sarcoplasmic reticulum calcium channel ryanodine receptor (*RYR1*) and the giant elastic filament protein titin (*TTN*) (Böhm et al., 2010; Ceyhan-Birsoy et al., 2013; Claeys et al., 2010; Mejaddam et al., 2009; Nicot et al., 2007; Wilmshurst et al., 2010). Similar to *Smyd1* CKO mice, humans with CNM caused by *BIN1* mutations often present with hypotrophy, dense oxidative staining in the vicinity of the central nuclei, myofibrillar disorganization and a nonradial sarcoplasmic reticulum pattern. Unlike the *Smyd1* CKO, type I fiber predominance is common (Jungbluth and Gautel, 2014; Nicot et al., 2007; Toussaint et al., 2011). Comparable to the *Smyd1* CKO, some individuals with CNM caused by *RYR1* mutations exhibit regional myofibrillar disorganization and hypotrophy (Bevilacqua et al., 2011), but, unlike the *Smyd1* CKO, type I fiber predominance is common (Wilmshurst et al., 2010). Humans with CNM caused by *TTN* mutation present with multiple internal nuclei, type I fiber predominance, hypotrophy and endomysial fibrosis (Ceyhan-Birsoy et al., 2013) – symptoms collectively dissimilar to that observed for the *Smyd1* CKO. The phenotype observed in the *Smyd1* CKO is most similar to a subset of clinical CNM categorized as ‘congenital myopathy with prominent nuclear internalization and large areas of myofibrillar disorganization’ (Romero, 2010). A small subset of individuals with CNM also present with cardiomyopathy. CNM with cardiomyopathy is linked to *SPEG* and *DNM2* mutations, but, in many cases, the genetic cause is unknown (Agrawal et al., 2014; Al-Ruwaishid et al., 2003; Gal et al., 2015; Gospe et al., 1987; Verhiest et al., 1976). Because SMYD1 is a crucial regulator of both heart and skeletal muscle development, it is a good candidate for the genetic lesion in cases in which myofibril disarray presents in both skeletal and cardiac myocytes.

Despite its important functions in striated muscle, *SMYD1* mutations have not been identified in any patient with congenital myopathy. It is known that SMYD1 has a crucial role in heart development (Gottlieb et al., 2002); thus, humans with *SMYD1* mutations would likely exhibit congenital heart defects. No clinical cases of congenital heart defects arising from *SMYD1* mutations have been reported, but one report found single-nucleotide polymorphisms in exon 6 associated with hypertrophic cardiomyopathy (Abaci et al., 2010). Thus, human *SMYD1* loss-of-function mutations might be so detrimental to heart development that, like for mice, embryonic survival is compromised and myopathy is not assessed.

Loss of SMYD1 was more detrimental to males than to females. *Smyd1* CKO males exhibited a greater percent loss of strength, muscle mass and body weight than females. Likewise, CKO females exhibited fewer fibers with central nuclei (19% in males vs 9% in females) and less reduction in myofiber diameter. These observations suggest that loss of SMYD1-induced myopathy has a greater impact on males than females. Other than in X-linked myotubular myopathy (XLMTM), to our knowledge, the effect of sex on the severity of CNM disease has not been reported. As for prevalence, *DNM2* and *BIN1* mutations seem to equally affect both sexes (Böhm et al., 2010; Jeub et al., 2008). *TTN* mutations were only reported in males (Ceyhan-Birsoy et al., 2013). *RYR1* mutations were reported for both males and females (Bevilacqua et al., 2011; Wilmshurst et al., 2010). Our data raise the possibility that gender-specific differences in autosomal CNM have gone unnoticed.

Loss of SMYD1 primarily produced centralized nuclei and hypotrophic fibers in fast-twitch muscles; the soleus was largely unaffected. This fiber-type difference was also apparent in real-time PCR assays where alterations in gene expression were only observed for the tibialis anterior. The pathological differences between fast- and slow-twitch muscles are unlikely to be caused by differences in *Smyd1* expression because approximately equivalent levels of the SMYD1 protein were observed in both tibialis anterior and soleus. Furthermore, SMYD1 exhibited similar sarcomeric localization in both type I and II fibers. Therefore, SMYD1 might have discrete functions in fast- and slow-twitch fibers, and the precise function of SMYD1 in type I fibers remains unknown.

The lack of an obvious phenotype in slow-twitch muscle could be the result of differential expression of a SMYD1 methylation target or SMYD1-interacting molecule. We found no differences in gene expression in control versus *Smyd1* CKO soleus for any of the genes assayed by real-time PCR. Thus, slow-twitch fibers might lack one or more SMYD1-interacting co-regulators or downstream-acting transcription factors. We tested the hypothesis that increased expression of other SMYD family members compensates for the absence of SMYD1 in the soleus; however, the results of these experiments showed that this was not the case. Expression of *Smyd2*, *3*, *4* and *5* was unaffected in the soleus. In the tibialis anterior, the only SMYD family member with altered expression was *Smyd2* and it was downregulated. SMYD2 contributes to titin stability and maintenance of the I-band by methylating HSP90 (Donlin et al., 2012). Thus, defects in fast-twitch, but not slow-twitch, muscles could be due to a synergistic effect of absence of SMYD1 and reduction of SMYD2.

*Smyd1* CKO tibialis anterior exhibited increased numbers of oxidative fibers as assayed by NADH-TR staining. Because there was no increase in abundance of type I fibers in the tibialis anterior, these data most likely reflect an increase in type IIa and IIx fibers. Overexpression of *Myog* promotes switching from glycolytic to oxidative metabolism without altering fiber type (Hughes et al., 1999). Furthermore, *Myog* is expressed to a greater level in oxidative fibers (Voytik et al., 1993). Thus, the increased prevalence of oxidative fibers in the *Smyd1* CKO could explain why *Myog* was upregulated. *Myog* upregulation in this situation might have more to do with increased oxidative metabolism rather than regeneration, which was almost completely absent. Note that, although CKOs exhibited increased numbers of oxidative fibers, central nuclei were most prevalent in glycolytic fibers. Thus, the presence of a central nucleus is not the direct cause of the difference in fiber type, but we cannot exclude the possibility that the oxidative fibers had a central nucleus at some point in the past.

The most striking defect identified by TEM was myofibrillar disorganization. We suspect that the areas of myofibrillar disorder must not be large enough to cause fiber degeneration, but might still promote expression of contractile genes in order to repair the defects. These data support findings in zebrafish, suggesting that SMYD1 is important for sarcomere assembly and stability (Just et al., 2011; Li et al., 2013; Tan et al., 2006). Knockdown of *smyd1a* and *b* in zebrafish embryos severely impairs sarcomerogenesis by 24 hpf, leaving thick and thin filaments in complete disarray. Affected myofibers also exhibited centrally located nuclei. Thus, it was concluded that SMYD1 is required for myofibril organization during myofiber maturation (Tan et al., 2006). Our data are consistent with these observations in that we observed myofibrillar disorganization, albeit not to the extent seen in zebrafish mutants. These differences in phenotype could be due to timing of *Smyd1* ablation. Zebrafish mutants or morphants lose *Smyd1* expression from the myoblast stage, whereas our CKO eliminates *Smyd1* in myofibers perinatally.

The mechanism(s) through which SMYD1 contributes to myofibrillogenesis is largely unknown. SMYD1 colocalizes with nascent myosin in immature sarcomeres and then with the M-line protein myomesin in mature sarcomeres (Just et al., 2011). Thick-filament chaperones *hsp90a1* and *unc45b* are upregulated in zebrafish when *smyd1a/b* expression is reduced (Just et al., 2011; Li et al., 2013). These observations implicate SMYD1 in the regulation of myosin folding, degradation and assembly into sarcomeres. Morpholino-mediated downregulation of zebrafish *smyd1b* led to decreased myosin protein without affecting mRNA levels (Li et al., 2013). In contrast, our data from *Smyd1* CKO mice revealed upregulation of all MyHC isoform genes and concomitant increases in MyHC proteins, with the exception of slow MyHC/Myh7. Because many other muscle developmental genes were upregulated in the *Smyd1* CKO, we suggest that increased expression of MyHC and other muscle genes is a repair response due to myofibril damage or failure to completely mature myofibers.

CNM is considered to be a disease of the triads, the anatomical site of excitation-contraction coupling within a myocyte (Jungbluth and Gautel, 2014). In fact, most of the genes linked to CNM affect the triad either directly or indirectly through importance in membrane curvature or endocytosis. We did not observe structural triad defects, but found reduced numbers of triads in the *Smyd1* CKO tibialis anterior and broad alterations in expression of triad-associated genes. Reduced triad numbers reflect lack of a fully developed triad network, which should envelope each sarcomere. Thus, in *Smyd1* CKO myocytes, there is less triad surface area per sarcomere, which would adversely affect excitation-contraction coupling. Reduced triad numbers might reflect impaired fiber maturation. Alternatively, centrally located nuclei might affect the overall architecture of the intermyofibrillar network. Architectural abnormalities in the intermyofibrillar network could result in decreased numbers of triads per Z-line junction as assayed in this study.

Because SMYD1 is a muscle-specific histone methyltransferase, we suspected that the CKO phenotype would, at least in part, result from altered epigenetic regulation/transcription of genes known to cause CNM. However, reduced mRNA levels were only observed for *Ptpla* (∼60% decrease). *Ptpla* (also known as *HACD1*) encodes an enzyme required for elongation of very-long-chain fatty acids (Ikeda et al., 2008), which contributes to the architecture of curved membranes. Triads are structures comprising highly curved membranes and CNM often results from triad defects – thus, establishing a molecular link between *Ptpla* and CNM. Reduced expression of *Ptpla* could impair the construction of triads and lead to reduced triad numbers, as observed in the *Smyd1* CKO.

SMYD1 represses transcription when tethered to DNA, via recruitment of co-repressors to its MYND domain (Gottlieb et al., 2002). Thus, SMYD1 might be a repressor of muscle developmental gene expression. This could explain why SMYD1 is exported from the nucleus to the cytosol during myoblast fusion (Berkholz et al., 2014; Nagandla et al., 2016; Sims et al., 2002) and the reason why muscle development genes were upregulated in the *Smyd1* CKO. The only transcription factor known to interact with SMYD1 is the muscle-specific activator skNAC (Sims et al., 2002; Yotov and St-Arnaud, 1996). Most *skNAC*-null mice die during embryogenesis, owing to heart defects; those that survive exhibit muscle defects, including reduced mass and impaired regeneration, but not CNM (Park et al., 2010). Differences in muscle phenotypes between *skNAC*-null and *Smyd1* CKO mice illustrate non-redundant functions for SMYD1 and skNAC. skNAC is a transcription factor, whereas SMYD1 is both a co-regulator and sarcoplasmic effector of myofibrillogenesis.

In summary, elimination of SMYD1 from skeletal muscle produces a myopathy characterized by increased internal nuclei, hypotrophy, myofibrillar disarray and weakness. Defects primarily manifest within type II fibers, illustrating fiber-type differences in SMYD1's biological activities. Furthermore, our data reveal a gender-specific penetrance in phenotype that could relate to clinical cases where men are more severely affected than women. Collectively, our results suggest that loss of SMYD1 affects myofiber maturation and implicate protein methylation in myofibrillar organization.

## MATERIALS AND METHODS

### Animals

*Smyd1^flox/flox^* mice were developed in collaboration with Dr Haley Tucker (University of Texas, Austin, TX) (Rasmussen et al., 2015). *Smyd1^+/−^* mice were generated by crossing *Smyd1^flox/flox^* to the maternal deleter *Tg(Sox2-cre)* (Hayashi et al., 2003) (Jackson Laboratory, stock #8454). *Rosa26^YFP/YFP^* and *Myf6^cre/+^* mice were obtained from the Jackson Laboratory (stock #7903 and #10528, respectively). Mice for experimental analysis were obtained from the following cross: *Myf6^cre/+^; Smyd1^+/−^*×*Smyd1^flox/flox^; Rosa26^YFP/YFP^*. Controls were *Myf6^cre/+^; Smyd1^flox/+^; Rosa26^YFP/+^*. CKOs were *Myf6^cre/+^; Smyd1^flox/−^; Rosa26^YFP/+^*.

The following primers were used for genotyping: Cre-F: 5′-GCCACCAGCCAGCTATCAACTC-3′, Cre-R: 5′-TTGCCCCTGTTTCACTATCCAG-3′, Smyd1-F: 5′-TCATGAGATGGGCATGAGCC-3′, Smyd1-R1 5′-GCATACGCACATGTGCTCGC-3′, Smyd1-R2: 5′-CTCACTTGCGTCCCAGTACTTG-3′. Smyd1-F/R1 identifies wild-type and flox alleles (432 bp and 552 bp, respectively). Smyd1-F/R2 identifies the null allele (∼500 bp). All experimental procedures involving mice were approved by the Institutional Animal Care and Use Committee of the University of Houston.

### Strength and motor coordination tests

Strength was assayed by a grip strength test. The device consisted of a scale attached to a wire mesh. The scale was immobilized and grip strength was measured by allowing the mouse to grasp the wire mesh with all four paws and then pulling the tail with increasing force until it releases its grip. The test was repeated three times per mouse with a 15 s interval between tests. Data reported is the maximum pull of the three trials.

Strength was also assayed by an inverted grid hang test (Deacon, 2013). This device consists of a wire grid suspended 12 inches above a soft surface. The mouse was placed on the wire grid, which was then inverted such that the mouse was hanging from the grid upside down. Data was time to fall. The trial was stopped if the mouse did not fall after 5 or 8 min for males and females, respectively.

Strength, endurance and motor coordination was assayed by a rotarod test. The test consisted of four trials per day for two consecutive days with 15 min intervals. The rotarod apparatus (Med Associates Inc. ENV-577M) was programmed for continual acceleration from 4 to 40 rpm over 5 min. Data recorded were either the time that had elapsed when the animal fell from the rotarod or when the animal completed two 360° rotations, whichever occurred first.

### Body composition

Body composition (% fat and lean mass) was calculated by MRI using an Echo MRI (Echo Medical Systems, Houston, TX).

### Evans blue dye uptake

To assay sarcolemmal integrity, one group of mice was not exercised, whereas a second group received a single treadmill exercise regimen of 30 min running at 12 m/min on a 5° incline. Evans blue dye (5 μl of 1% solution in PBS/g body weight) was given by intraperitoneal (IP) injection 30 min post-exercise. The non-exercised group received Evans blue dye at the same time. Tibialis anterior muscle was collected 24 h post-injection as described below. Evans blue dye uptake was assayed in cryosections by far-red fluorescence.

### Tissue collection

Muscles were dissected in PBS immediately after euthanasia. Excess PBS was removed by blotting on a paper towel. Muscles were then weighed in pairs using an analytical scale (Denver Instrument, model# SI-114). For RNA or protein extraction, tissues were snap-frozen in liquid nitrogen and stored at −80°C. For histology (H&E staining), tissues were fixed by overnight immersion in Bouin's fluid. For immunofluorescence, Evans blue dye fluorescence and NADH-TR staining, tissues were snap-frozen in liquid-nitrogen-cooled isopentane and stored at −80°C until preparation of cryosections.

### Real-time PCR

Total RNA was extracted from muscle using TRIzol reagent according to the manufacturer's protocol (Life Technologies, Carlsbad, CA). mRNA levels were measured using TaqMan gene expression assays with 6-carboxyfluorescein (FAM)-labeled probes from Applied Biosystems (Life Technologies) (Mm00437457_m1 for *Bin1*, Mm00514582_m1 for *Dnm2*, Mm01282275_m1 for *Mtm1*, Mm01184733_m1 for *Mtmr14*, Mm01332489_m1 for *Myh1*, Mm01332564_m1 for *Myh2*, Mm01332463_m1 for *Myh3*, Mm01332518_m1 for *Myh4*, Mm01319006_g1 for *Myh7*, Mm01329494_m1 for *Myh8*, Mm01203489_g1 for *Myod1*, Mm00446195_g1 for *Myog*, Mm01354484_m1 for *Pax7*, Mm01729860_m1 for *Ptpla*, Mm01175211_m1 for *Ryr1*, Mm00477663_m1 for *Smyd1*, Mm00660598_m1 for *Smyd2*, Mm00510201_m1 for *Smyd3*, Mm00662052_m1 for *Smyd4* and Mm00523415_m1 for *Smyd5*) and custom oligos for *Pax3* (Pax3-F: 5′-CCAGAGGGCGAAGCTTACC-3′, Pax3-R: 5′-GTTGATTGGCTCCAGCTTGTTT-3′, Pax3-Probe: 5′-TCTGGTTTAGCAACCGCCGTGCA-3′, Biosearch Technologies, Petaluma, CA). The level of *Gapdh* mRNA was used for normalization. The PCR was run using an ABI Prism 7900HT thermocycler and SDS2.1 software (Applied Biosystems). Data were analyzed by the comparative ΔΔCT method.

### Western blotting

Western blot assays were carried out using total cellular protein. Frozen muscle was homogenized in T-PER buffer (20 ml/g of tissue) (Life Technologies) plus protease and phosphatase inhibitors according to the manufacturer's instructions. Primary antibodies were against Smyd1 (Santa Cruz, cat# sc-79080), myosin heavy chain (MyHC) [Developmental Studies Hybridoma Bank (DSHB), clone MF-20], slow MyHC (DSHB, clone A4.840) or β-tubulin (DSHB, clone E7-c). Membranes were visualized by chemiluminescence using SuperSignal West Pico Chemiluminescent Substrate (Thermo Scientific, Rockford, IL).

### Histological analyses

H&E staining was performed using a standard protocol. The percentage of myofibers with centralized nuclei was determined by manual counting. The number of fibers exhibiting centralized (internal) nuclei was divided by the total number of fibers in the tissue cross-section (approximately 2900 fibers/tibialis anterior and 700 fibers/soleus). Myofiber minimum Feret diameter was calculated using the measurement feature of NIS Elements software (Nikon Instruments, Tokyo, Japan). For soleus, 500 fibers were measured. For tibialis anterior, 72 fibers were measured at seven evenly spaced locations (total of 504 fibers).

NADH-TR staining was performed on 10-μm tibialis anterior cryosections by standard methods. The oxidative reaction was conducted for 15 min at room temperature followed by quenching with PBS. Slides were then stained with DAPI and coverslips mounted with VECTASHIELD HardSet Mounting Medium (Vector Laboratories, Burlingame, CA) to correlate oxidative staining peculiarities with the presence of centralized nuclei.

Immunofluorescence was performed on 10-μm cryosections by standard methods. 1× casein-10% normal serum-PBST was used for protein blocking and as the antibody diluent (10× casein stock solution and normal serum were from Vector Laboratories). All slides were stained with DAPI and coverslips mounted with Vectashield Hard Set mounting medium (Vector Laboratories). Antibodies were: goat anti-Smyd1 (Santa Cruz, cat# sc-79080), mouse anti-slow-MyHC (DSHB, clone A4.840) and mouse anti-embryonic-MyHC (DSHB, clone F1.652).

### Microscopy

Stereoimages (brightfield and fluorescence) were captured with a Leica MZ10F stereomicroscope and the extended depth-of-focus feature of LAS v3.7 software (Leica Microsystems, Wetzlar, Germany). Brightfield and epifluorescence images of tissue sections were obtained using a Nikon Ti-E inverted microscope equipped with a DS-Fi1 5-megapixel color camera (Nikon Instruments), a CoolSNAP HQ2 14-bit monochrome camera (Photometrics, Tucson, AZ) and NIS Elements software v4.13 (Nikon Instruments).

### Transmission electron microscopy

1- to 2-µm cubes (six cubes/animal) of tibialis anterior muscle were immersion fixed in 0.1 M sodium cacodylate buffer (pH 7.2) containing 2.5% glutaraldehyde and 20 mM calcium chloride. Tissues were post-fixed and heavy-metal-contrasted with potassium ferrocyanide, osmium tetroxide, thiocarbohydrazide, uranyl acetate and lead aspartate (Lafontant et al., 2013). After dehydration through an acetone series, tissues were embedded in Embed-812 resin (Electron Microscopy Sciences, Hatsfield, PA) and ultrathin sections (100 nm) were imaged on a Tecnai G2 Spirit BioTWIN (FEI Company, Hillsboro, OR) electron microscope. The abundance of triads was calculated by counting the number of triads per Z-line junction. At least 75 non-overlapping images were captured per animal at 26,500× from at least 15 individual fibers. Images were evenly spaced along the length of each fiber and no more than five images were captured per fiber.

### RNA-seq

Tibialis anterior total RNA (100 ng) from six control and six CKO mice was depleted of ribosomal RNA using the ScriptSeq Complete Gold kit from Epicentre (cat# SCL24EP) following the manufacturer's instructions. The 12 libraries were submitted at a 20 nM-multiplexed concentration for sequencing on the Illumina HiSeq platform at the DNA core facility at the University of Texas M. D. Anderson Cancer Center (Houston, TX). After adapters were trimmed from raw RNA-seq data, reads were aligned to the mouse genome using bowtie2 (Langmead et al., 2009) and tophat (Trapnell et al., 2009), allowing at most two mismatches. Reads that mapped to multiple locations in the genome were discarded. The abundance of each transcript was computed using cufflinks. The abundance of each transcript was expressed as FPKM (fragments per kilobase of transcript per million mapped reads). We used DESeq to obtain differentially expressed (*P*<0.05) genes between two conditions (Anders and Huber, 2010). Genes with less than three aligned read counts across all the samples were not considered. Enriched gene ontology terms were obtained using DAVID (Huang et al., 2009) with the mouse genome as the background set of genes. Robust *z*-score, which was computed for each gene from the FPKM values across all the replicates of the two conditions, was used to generate the heat maps. The dataset was deposited to the NCBI Gene Expression Omnibus (GEO) database under series accession number GSE71679 (http://www.ncbi.nlm.nih.gov/geo/query/acc.cgi?acc=GSE71679).

### Statistical analyses

For experiments with more than two experimental groups, quantitative data were subjected to one-way analysis of variance (ANOVA) using MedCalc v14.12.0 software (MedCalc Software bvba, Belgium). Rotarod data was analyzed by two-way ANOVA. If the ANOVA was positive (*P*<0.05), a post-hoc Student–Newman–Keuls test was performed for pairwise comparison of subgroups. For studies with two experimental groups, an independent samples Student's *t*-test was performed. A *P*-value of <0.05 was considered significant for all tests.
